# The Impact of Telehealthcare on the Quality and Safety of Care: A Systematic Overview

**DOI:** 10.1371/journal.pone.0071238

**Published:** 2013-08-19

**Authors:** Susannah McLean, Aziz Sheikh, Kathrin Cresswell, Ulugbek Nurmatov, Mome Mukherjee, Akiko Hemmi, Claudia Pagliari

**Affiliations:** eHealth Research Group, Centre for Population Health Sciences, The University of Edinburgh, Edinburgh, United Kingdom; University Hospitals of Geneva, Switzerland

## Abstract

**Background:**

Telehealthcare involves the use of information and communication technologies to deliver healthcare at a distance and to support patient self-management through remote monitoring and personalised feedback. It is timely to scrutinise the evidence regarding the benefits, risks and costs of telehealthcare.

**Methods and Findings:**

Two reviewers searched for relevant systematic reviews published from January 1997 to November 2011 in: The Cochrane Library, MEDLINE, EMBASE, LILACS, IndMed and PakMed. Reviewers undertook independent quality assessment of studies using the Critical Appraisal Skills Programme (CASP) tool for systematic reviews. 1,782 review articles were identified, from which 80 systematic reviews were selected for inclusion. These covered a range of telehealthcare models involving both synchronous (live) and asynchronous (store-and-forward) interactions between provider and patients. Many studies showed no differences in outcomes between telehealthcare and usual care. Several reviews highlighted the large number of short-term (<12 months) feasibility studies with under 20 participants. Effects of telehealthcare on health service indicators were reported in several reviews, particularly reduced hospitalisations. The reported clinical effectiveness of telehealthcare interventions for patients with long-term conditions appeared to be greatest in those with more severe disease at high-risk of hospitalisation and death. The failure of many studies to adequately describe the intervention makes it difficult to disentangle the contributions of technological and human/organisational factors on the outcomes reported. Evidence on the cost-effectiveness of telehealthcare remains sparse. Patient safety considerations were absent from the evaluative telehealthcare literature.

**Conclusions:**

Policymakers and planners need to be aware that investment in telehealthcare will not inevitably yield clinical or economic benefits. It is likely that the greatest gains will be achieved for patients at highest risk of serious outcomes. There is a need for longer-term studies in order to determine whether the benefits demonstrated in time limited trials are sustained.

## Introduction

There is considerable international interest in the potential of telehealthcare to improve the convenience, quality, safety and cost-effectiveness of healthcare [1], [2]. This has accompanied the realisation that traditional models of health service delivery may be unable to cope with future levels of chronic disease in ageing populations [3], [4], [5], [6]. Economic pressures on health systems also call for solutions that will keep such patients out of hospital and in their own homes for as long as possible [7], [8]. Achieving widespread telehealthcare is a key objective of the ‘Digital Agenda for Europe’ and is a key feature of the UK’s NHS Information Strategy [9], [10], [11]. It has also become a strategic priority for major healthcare providers in the United States such as the Veterans Administration, which aims to offer daily telehealthcare to over 28,000 of its members by 2014 [12].

We have previously defined telehealthcare as “the provision of personalised health care over a distance” [13]. By personalised we refer to the use of information and communication technology (ICT) as a medium for enabling professional-patient interaction, in contrast to more passive information delivery or monitoring without feedback. Telehealthcare may be synchronous (real-time), as in video-conferencing or telephone, or asynchronous, as with email and other store-and-forward methods.

Despite the high levels of interest in telehealthcare, and considerable industry hype surrounding its potential to reduce healthcare costs and improve patient outcomes, there has not always been adequate scrutiny of the scientific evidence base underpinning it. We were commissioned by the National Health Service Connecting for Health Evaluation Programme to review this evidence, as part of a wider review on the quality and safety of eHealth. The first part of our systematic overview was published in 2011, focusing on electronic health records and computer decision support systems [14], [15], [16]. This follow-on work involved identifying, critically reviewing, summarising and interpreting the evidence regarding the impact of telehealthcare on the quality and safety of care [17].

## Methods

### Overview of Methods

In keeping with our related systematic overview [14], we chose to conduct a systematic review of systematic reviews in order to generate a high level synthesis of the evidence that offered the potential to inform national and international policy deliberations with regard to telehealthcare applications.

### Developmental Work

There are many terms used to describe the remote delivery of care: “telehealth”, “telecare”, “telemedicine” and “home telemonitoring” are among the most often used. We reviewed many definitions of these terms and placed them within a conceptual framework [17], [18]. We have chosen to use the term “telehealthcare” to describe the interaction, over a distance, between a patient and a health care professional. S*ynchronous* models of telehealthcare involve real-time interactions, (e.g. telephone or video-conferencing with or without physiological monitoring), while *asynchronous* telehealthcare models use store-and-forward methods, such as Short Message Service (SMS), email consultation or monitoring of symptoms and signs through networked devices that collect and upload data at intervals. We were interested in both synchronous and asynchronous models of telehealthcare.

While synchronous methods of consulting are often used to overcome barriers of physical distance, strategies for the management of long-term conditions are increasingly focused on supporting patient self-care through enabling remote measurement of symptoms, either through questionnaires or through monitoring devices which transmit data (e.g. blood glucose, blood pressure, weight, electrocardiogram) via telephone, Internet or mobile network to one or more healthcare professionals who review the data and then use their clinical judgement to make recommendations and deliver patient-specific feedback to the individual [13].

Telehealth models involving automated, algorithm-driven feedback in response to self-monitoring data (as in self-testing of blood pressure with numerical feedback or computer-generated guidance) were not included within this review. Also outside of the scope of this review were the use of distance technologies to facilitate communication between healthcare professionals (often, although not exclusively, referred to as “telemedicine”), medical e-learning or one-way online health information interventions/portals.

### Search Strategy

We searched The Cochrane Library, MEDLINE, EMBASE, LILACS, IndMed and PakMed for systematic reviews published from January 1997 to November 2011 to identify those investigating the impact of telehealthcare interventions on the quality and safety of care. We drew on the comprehensive list of Medical SubHeadings (MeSH) and free text terms covering the concepts of “quality” and “safety” which we had developed for our earlier overview [14]. These sets of terms were combined with MeSH and key words related to the concept of “telehealthcare” and with the methodology terms “systematic review or meta-analysis” (see S1 Supplement 1 Search Strategy). We also searched Google Scholar and the University of York’s PROSPERO database for further published and unpublished material, using modified search strategies.

### Selection of Systematic Reviews

At least two reviewers (SM, UN or KC) independently assessed the retrieved studies for inclusion against our inclusion/exclusion criteria and reached agreement through discussion. If agreement could not be resolved in this way a further reviewer (CP) arbitrated.

### Inclusion Criteria

The only study design that was eligible was that of a systematic review. We identified these as studies referring to themselves as systematic reviews in the title or abstract, or studies which used systematic review methodology on closer inspection of the methods section. We did not consider the different types of study designs included within the systematic reviews, as these were very varied. The interventions described by the review had to fall within our definition of telehealthcare as personalised feedback delivered by a healthcare professional in response to patient-specific data via ICT.

### Exclusion Criteria

We excluded diagnostic or treatment advice from subject experts (e.g. telepathology/teledermatology) and conferencing between healthcare professionals. Also excluded were examples in which health professionals were gaining mobile access to records, evidence or computerised decision support without a patient-to professional-interaction. Online forums and peer groups were excluded unless there was evidence that a healthcare professional was making personalised recommendations to the forum to support individual patients. Systems that provided only automated, computer-driven, feedback in response to patient self-monitoring data were also excluded.

### Critical Appraisal of Systematic Reviews

Two reviewers (SM, KC) independently assessed the quality of each of the included systematic reviews using the Critical Appraisal Skills Programme (CASP) tool (see S2 Supplement 2 CASP Score Tool) [19]. Reviewers discussed areas of disagreement and resolved these through discussions to agree moderated scores. In cases where it was not possible to reach agreement, a third reviewer arbitrated (CP). With the assistance of UN, MM and AH, none of the authors were involved in data extraction or quality appraisal of their own previous work.

### Data Synthesis

We abstracted key information regarding telehealthcare interventions from the body of systematic reviews, having piloted several data-extraction templates, concentrating on different clinical areas of practice and categories of evidence according to our conceptual frameworks. We systematically extracted data on the key benefits and risks of telehealthcare. This involved identifying benefits in clinical endpoints to patients, then benefits in terms of patients’ quality of life, and other benefits for patients. Benefits for professionals and benefits for the healthcare system were also identified and any other benefits. Finally, we reviewed risks for patients, professionals or the healthcare system.

## Results

### Overview of the Evidence

Searches undertaken as part of our previous work identified 1716 potentially relevant reviews from which we selected 41 systematic reviews which focused on telehealthcare [17]. Re-running the searches to cover the period up to and including November 2011 resulted in a total of 81 systematic reviews meeting our inclusion criteria (see S3 Supplement 3 PRISMA Figure).

The 81 systematic reviews synthesised evidence from 2,396 original articles, excluding duplicates, including 579 randomised controlled trials (see S4 Supplement 4 Studies Figure). The focus of each review was variable. Most focused on a specific disease area. However, there were also reviews focusing on telehealthcare in the frail elderly [20], [21], patient satisfaction [22], [23], [24], telephone consultation [25], [26], [27], video-conferencing [28], [29], rural telehealthcare [30] and telehealthcare in Asian countries [31]. In this article, when we use the term “review” we are referring to the included 80 systematic reviews that have been quality appraised; when we discuss “studies” we are referring to the studies included within the reviews themselves.

Telehealthcare was involved in the ongoing management of various long-term conditions and there were seven reviews on this subject [21], [28], [32], [33], [34], [35], [36]. There were eight reviews which specifically examined the role of telehealthcare in caring for diabetic patients [37], [38], [39], [40], [41], [42], [43], [44], six reviews solely on chronic heart failure [45], [46], [47], [48], [49], [50] and eight reviews on mental disorders including drug addiction [51], [52], [53], [54], [55], [56], [57], [58], [59]. There were two reviews (published in three reports), concerning asthma [60], [61], [62] and two on chronic obstructive pulmonary disease (COPD) [63], [64]. In such systematic reviews, telehealthcare was used to monitor the patient’s condition; frequently by relaying questions about symptoms such as breathlessness and prompting measurement of other parameters such as weight and blood glucose. Telehealthcare was also used to deliver advice to the patient, either to change treatments or to modify the frequency of monitoring. In the area of mental health, telehealthcare was used to deliver cognitive behavioural therapy from a distance, often via videoconferencing or telephone, but there were also trials of computer-based cognitive therapy delivered as part of a package of therapist-guided interventions.

### Quality of Evidence

The evidence amassed was generally of moderate quality, with the majority of studies scoring between 15 and 25 out of 30 according to the CASP criteria (see S5 Supplement 5 Summary Table [19]). Several reviews highlighted the predominance of small pilot-like studies with fewer than 20 subjects [20], [22], [65], [66]. Such studies were mainly useful for demonstrating the feasibility of the technology. Some of the studies included in the reviews did not report power calculations, placing them at risk of Type II errors [66]. Often reviews contained studies that were not randomised or that were designed as observational studies, pilot studies or pre-post studies, limiting the weight that can be given to their conclusions [21], [22], [23], [24], [26], [28], [29], [31], [33], [34], [35], [36], [38], [40], [41], [42], [43], [48], [49], [52], [58], [67], [68], [69], [70], [71], [72], [73], [74], [75], [76], [77]. A number of reviews did not clearly detail the study designs of all the included studies [20], [30], [32], [51], [60], [78], [79], [80], [81], [82], [83], [84], [85], [86]. In one review the number of studies included was not clear [60].

### Clinical Outcomes

#### Surrogate endpoints

Many government and industry claims made around the benefits of telehealthcare include reduction of mortality, morbidity, frequency of hospitalisations and number of bed days of care. We searched for these ‘hard’ clinical endpoints, but we found instead that many reviews of telehealthcare reported ‘surrogate’ endpoints such as changes in mean HbA1c, blood pressure and lipid profile [37], [38], [39], [40], [41], [42], [43], [44]. The advantage of evaluating surrogate endpoints is that smaller, shorter trials may be undertaken. The authors of such studies often infer that as it is “known” that, for example, improving HbA1c improves diabetes related hard clinical outcomes such as the incidence of myocardial infarction and strokes, there is no need to repeat that finding [87], [88]. We often encountered a situation where a result was reported as of statistical significance, but where its clinical significance was equivocal [22], [38], [51], [53], [65], [68], [71], [78], [84], [89].

The reviews yielded some examples of studies showing small improvements in surrogate endpoints for long term conditions. In diabetes, there were small but statistically significant pooled decreases in HbA1c - In Type 1 diabetes (−0.4% difference, 95% C.I. 0.0 to −0.8) [40] and Type 2 diabetes (−0.6% in HbA1c [39]), (weighted mean difference, −0.2%, 95% C.I. −0.4 to −0.1 [41]). One review did not pool results but reported that a reduced HbA1c was demonstrated in six trials [42]. Another two reviews reported that there was no difference in HbA1c in a pooled statistic from 19 randomised controlled trials (weighted mean difference −0.1%, 95% C.I. −0.4 to 0.2) [43] and in a pooled statistic from seven trials (weighted mean difference of −0.4%, 95% C.I. −0.9 to 0.1). Overall, the data suggested that these interventions had at best only modest effects on blood glucose control.

Two systematic reviews focused on home management of blood pressure by patients with hypertension [78], [90]. In one review of 22 trials there was a small change in the pooled mean systolic BP of −2.6 mmHg (95% C.I. −4.2 to −1.0) and in the pooled mean diastolic BP of −1.7 mmHg (95% C.I. −2.6 to −0.8) [78]. In another review, office blood pressure improved significantly more in patients randomised to home telemonitoring: systolic −5.7 mmHg (95% C.I. −7.9 to −3.4), diastolic −2.8 mmHg (95% C.I. −3.9 to −1.62) pooled across 11 studies. However, the differences in ambulatory blood pressure were less marked: systolic: −2.3 mmHg (95% C.I. −4.3 to −0.2); diastolic 1.4 mmHg, no difference (95% C.I. −3.6 to 0.8) [90] Again, these differences are small, raising questions about their clinical relevance.

In summary, there were some small statistically significant improvements reported in surrogate endpoints but these were not consistent across all studies and unlikely to have significant clinical impact.

#### Hard clinical endpoints

It is important to see if any improvements in surrogate endpoints can be translated into improvements in hard clinical endpoints, such as mortality.

The strongest evidence in this respect is in relation to chronic heart failure. There were several studies looking at mortality in the context of telehealthcare for heart failure patients [45], [46], [49], [50]. One review searched for studies which compared home telemonitoring to usual care and found reduced all-cause mortality [49]. The authors pooled data from five randomised controlled trials and one observational study. The pooled relative risk was 0.6 (95% C.I. 0.5 to 0.8). In another review which specifically only included trial arms that compared telemonitoring to usual care and excluded arms with nurse only support or telephone support, reviewers also found a reduction in all-cause mortality based on 10 studies, relative risk 0.8 (95% C.I. 0.6 to 1.0) [45] A Cochrane review described the telehealthcare intervention as a multi-disciplinary care model typically involving ICT and which may include self-monitoring and education [46]. It classified interventions as “structured telephone support” if the monitoring and/or self-care management was delivered using simple telephone technology (although data may have been collected and stored by a computer) and “telemonitoring” if there was digital/satellite/broadband/wireless or Bluetooth transmission of physiological data. The Cochrane reviewers further defined the intervention as having to have been initiated by a healthcare professional and delivered to patients with chronic heart failure in the community as the only aftercare intervention, without home visits or intensified clinic follow-up. The comparison was usual care without intensified attendance at cardiology clinics or clinic-based chronic heart failure disease management programme or home visiting. In the pooling of all-cause mortality data from 15 studies structured telephone support resulted in a non-significant relative risk of 0.88 (95% C.I. 0.76 to 1.01) [46] and telemonitoring resulted in a significant relative risk of 0.66 (95% CI 0.54 to 0.81). And so overall it is probable that mortality from heart failure can be reduced with telemonitoring, but not with structured telephone support.

There were two reviews that reported on clinical endpoints by meta-analyses of randomised controlled trials of home-based, secondary rehabilitation programmes for coronary artery disease following acute myocardial infarction or coronary artery bypass graft [70], [91]. The first review by Clark et al [91], did not pool studies according to the intervention (classified as paper-based, electronic, telephone or home visit) but according to the setting (hospital- or home-based rehabilitation) and outcome (all-cause mortality, cardiovascular events, quality of life and risk factors). This makes it very difficult to draw conclusions as to the usefulness of the different interventions as “home-based rehabilitation” could involve a manual, the telephone and/or home visits. However, in pooling six studies with the outcome of mortality there was no difference between home-based interventions versus in-hospital cardiac rehabilitation. In the second review by Dalal et al [70], home-based cardiac rehabilitation was defined as a structured programme with clear objectives for the participants, including monitoring, follow-up, visits and telephone calls, and this was compared with centre based cardiac rehabilitation where a supervised group based in a hospital or community setting underwent rehabilitation. Dalal et al found no difference in mortality (relative risk 1.3 95% C.I. 0.7 to 2.7) [70] and no significant difference in cardiovascular events (stroke, transient ischaemic attacks and heart failure). There was also no difference in secondary prevention of coronary heart disease mortality with a telephone-mediated health behaviour change programme [92], [93].

A review of patients having telephone follow-up after cancer, compared to those having a face-to-face follow-up assessment, also showed no differences in mortality [83]. The telephone follow-up consisted of an interview carried out by a healthcare professional and patients were given a choice of using the telephone as part of their package of care. This review included 11 articles and described two trials where recognition of symptoms over the phone versus a face-to-face appointment was said to be equal: one trial was in breast cancer and the other was for urological symptoms. However, in these trials it was not clear to what extent the symptoms were recognised and assessed over the phone, nor was it apparent what the threshold for having a face-to-face appointment was following a phone assessment, nor whether such an appointment would be with a doctor or nurse or feature additional tests such as ultrasound.

The reviews of interventions for mental health investigated a variety of different clinical endpoints including diagnostic and symptom scores. Computerised cognitive behavioural therapy, with health professional oversight, had a positive impact in major depression according to the statistical measure used, but effects for social phobia panic disorder and generalised anxiety disorder were non-significant [94]. Telephone therapy for mild depression also improved symptom scores in one small study that was included in another review [53]. A review of Internet-based programmes for anxiety and depression in children and adolescents found symptom reduction and improvement in diagnostic ratings in six of the eight evaluation studies (three randomised controlled trials, two non-randomised controlled studies, two pre-post studies and one other study). There were two Internet-based programmes both supported by healthcare professionals either within the medical setting or remotely by email or telephone, who motivated users and supported their use of the programme, but no details of supervision for the other programmes were given [52]. Another review of computerised cognitive behavioural therapy for the prevention and treatment of depression and anxiety in children and adolescents included five randomised controlled trials and five case series and found that the percentage of participants meeting the diagnostic criteria for depression fell from 100% at the start of treatment to 30–78% post-treatment with the intervention. This review reported reductions in diagnostic severity, numbers of co-morbid diagnoses and anxiety and depressive symptoms [58]. However another review reported that only four out of eight studies showed a significant symptom reduction when compared to a control group [59]. Therefore, for mental health it seems that telehealthcare can result in small, significant benefit in symptoms.

Synchronous telehealthcare, such as video-conferencing, was suitable for the delivery of some rehabilitation techniques after stroke and spinal cord injury [75], [84]. However, conclusions regarding the effectiveness of telerehabilitation are premature as only 13 of the 56 included experimental studies involved a group of more than 20 patients, and not all of these studies were of robust design [75].

Several reviews commented that one advantage of telerehabilitation following stroke is that patients can access rehabilitation even if they live in a remote area. However, it was not clear from the presentation of the studies in the rehabilitation reviews whether the control groups were receiving standard outpatient rehabilitation or no rehabilitation [28], [73], [74]. There have been trials involving upper and lower limb rehabilitation, carer support and problem solving skills using video-conferencing and the Internet. In one review, there were four randomised controlled trials and four case series. It was not clear what the comparison groups were in the trials [73]. Another review included small studies of rehabilitation for a very diverse group of patients including: community elderly who had had falls or poor mobility, post total-knee replacement, post admission to geriatrics, knee pain, stroke, assessment for home care, multiple sclerosis, traumatic brain injury patients, post myocardial infarction, post cardiac surgery, spinal cord injury patients, speech and voice disorders, gait disorders, prosthetics, high care residents in residential care, and chronic pain. Outcomes were reported to be similar to those with face-to-face rehabilitation, with similar drop-out rates. There was some suggestion of decline in outcomes with longer term follow-up, but findings were inconsistent [74]. It was also reported that patients were more accepting of videoconferencing than staff [28].

In asthma, improvements in peak flow rates and symptoms were not consistently demonstrated with daily interactive monitoring and educational tools via the Internet [60], [61].

In COPD, there was no change in mortality with telehealthcare in a pooling from three trials (odds ratio 1.05 95% CI 0.63 to 1.75). The interventions in two of these three trials consisted of a web-based patient record with videoconferencing, or a web-based call centre with weekly phone calls and a co-ordinating case-manager. There was evidence from the third randomised controlled trial of a reduction in exacerbations, however, the intervention in this trial was well-resourced and complex including education and visits from nurses as well as telephone support [64]. There was also no change in mortality in this trial.

Therefore the evidence demonstrating improvements in hard clinical endpoints due to telehealthcare is modest and context-dependant. In general the evidence suggests that telephone only support models give results that are no better or worse than face-to-face. However, complex integrated interventions involving telemonitoring, education and additional support, potentially including home visits (which was not part of our telehealthcare definition) do have the potential to modestly improve outcomes.

### Health Service Utilisation

A decrease in the utilisation of healthcare services is often taken as an indicator of improved efficiency and quality in telehealthcare studies, for example through the replacement of face-to-face consultations with remote ones, or through reductions in hospital bed-days or emergency department visits as a result of better patient monitoring.

With regard to chronic heart failure: the Cochrane review of chronic heart failure reported a small significant reduction in all-cause hospitalisations for both structured telephone support compared with usual care (relative risk 0.9 (95% CI 0.9 to 1.0) and for telemonitoring compared with usual care (relative risk 0.9 (95% C.I. 0.8 to 1.0) [46]. In terms of chronic heart failure related hospitalisations, again both structured telephone support (relative risk 0.8, 95% C.I. 0.7 to 0.9) and telemonitoring (relative risk 0.8, 95% C.I. 0.7 to 0.9) demonstrated significant reductions [46]. Another review which focused on telemonitoring, pooled results for chronic heart failure from six studies for all cause hospital admission did not find a reduction for this outcome (risk ratio 1.0, 95% C.I. 0.9 to 1.0) [45]. However, for telemonitoring for chronic heart failure related hospital admission there was a significant reduction (risk ratio 0.7, 95% C.I. 0.6 to 0.9). A third review also found significant evidence of reduced all cause hospitalisations (incidence rate ratio 0.9, 95% C.I. 0.8 to 1.0) and chronic heart failure related hospitalisations (incidence rate ratio 0.8, 95% C.I. 0.7 to 0.9) [47]. This review pooled results for all kinds of remote patient monitoring in the form of either regular structured telephone contact between patients and healthcare providers, with or without a home visit, or technology assisted monitoring with transfer of physiological data and those with usual care consisting of patient visits to an outpatient clinic without additional phone calls [47]. A fourth meta-analysis reported significantly fewer all-cause hospitalisations with home telemonitoring in comparison with usual care, (relative risk 0.8, 95% C.I. 0.7 to 0.9) [49]. Another review reported decreased re-hospitalisation rates in patients with telemonitoring for heart failure [67]. Overall, it would appear that there is substantial evidence that telemonitoring reduces the risk of hospital admission in chronic heart failure. The evidence as to whether telephone support also reduces this risk is less clear cut.

In asthma there was significant reduction in hospitalisations over a 12 month period across four studies with a variety of telehealthcare interventions, including web-based diary and interaction with professionals, telephone calls from a nurse educator, telemonitoring with oversight for severe asthma and electronic diary with email or telephone follow up from a physician (risk ratio of 0.3 (95% C.I. 0.09 to 0.7) [61], [62]. These four studies were dominated by two studies in which the participants had severe and poorly controlled asthma and would appear to suggest that the more severe the patients’ condition the more they stand to benefit.

Two reviews reported reduced hospitalisations in COPD, again there was a variety of heterogeneous telehealthcare interventions pooled together, from simple repeated telephone support to complex interventions including education, telemonitoring and home some home visits [63], [64].

Two reviews of mixed chronic conditions concluded that the majority of included studies reported a significant decrease in emergency department visits, hospital admissions, and hospital length of stay for patients with heart and lung diseases [34], [36]. The first of these two reviews concentrated on home telemonitoring as an automated process for the transmission of data on a patient’s health status from home to the respective healthcare setting and included 65 studies but did not detail the interventions individually [34]. The second review was performed for the Canadian Agency for Drugs and Technologies in Health and classified interventions as either telemonitoring, involving data transmission and audio or video monitoring or telephone support, involving only telephone calls, they included 78 studies. For diabetes, heart failure and COPD they reported findings that there was evidence of reduced hospitalisations with both types of technology [36].

Similarly, a review of diabetes studies reported an average decrease in consultation time and decreased hospital admissions [43]. In this review the interventions were particularly targeted at monitoring clinical values and education. Diverse technologies were used for monitoring data, including palm-tops, glucometers, mobile phones and digital cameras, there was videoconferencing in some studies and others used web based disease management systems with multi-access capability. Another review of diabetes studies also reported a reduced number of patients hospitalised with telephone support, but found no studies of home-telemonitoring systems. [41].

Patterns of healthcare use changed significantly in some of the studies that compared face-to-face follow up for colorectal cancer with telephone follow up. Waiting times for a specialist appointment in one study dropped from 12 to 4 weeks and the incidence of patients failing to attend outpatient appointments decreased by 50%. Patients felt able to contact nurses for advice between appointments, which would also have implications for workload [83].

Telephone therapy did not make a statistically significant difference to hospital readmission for schizophrenia in one review of mental health [53].

One review reported reduced bed-days and emergency department visits for the elderly with chronic conditions, due to regular vital signs monitoring. However, it was not clear from the review how this monitoring was supervised [21].

A review of telephone follow-up initiated by a hospital-based health professional for post-discharge problems in patients discharged from hospital to home reported 11 studies examining health services related outcomes. Eight studies reported no difference, two studies reported fewer readmissions in the intervention group [25]. Of these two studies, one featured telephone follow-up for cardiac rehabilitation and the other, larger study (n = 242) was with telephone and computer decision support for people with chronic heart failure.

Overall, there appear to be many examples of telehealthcare reducing hospitalisations. Again there is a need for more context description to be given in studies regarding the specific nature of the interventions. We found some evidence that telemonitoring is probably more consistent in reducing hospitalisations than simple telephone support alone.

In general there was insufficient information included in the reviews to enable categorisation of patients by the severity of their illness. However, it would seem that where patients are more ill, e.g. with severe asthma rather than mild asthma, they stand to have more to gain from the interventions. This makes sense because applying the same relative risk reduction to those with a greater absolute risk of hospitalisation will result in a greater effect.

### Quality of Life

One review reported improvement in quality of life outcomes for some patients, for example in chronic heart failure and in asthma, but no improvement in diabetes [21].

The Cochrane review of heart failure reported that quality of life was a secondary outcome for 15 studies altogether, with nine studies reporting improvement in quality of life [46]. In another review of heart failure some studies included scores on SF-36 and the Minnesota Living with Heart Failure score, both of these improved, however the other included studies did not demonstrate an improvement in quality of life [45].

Several different quality of life scales were employed by the studies included in a review on the psychosocial outcomes of telephone-based counselling for adults with an acquired disability [84]. Most did not detect a significant difference following telecounselling compared with normal counselling.

There were no improvements in pooled quality of life in asthma with telehealthcare [62]: (mean difference 0.1, 95% C.I. 0.001 to 0.2) and none of the pooled nine studies individually measured a significant improvement in quality of life either. This may be because Juniper’s Asthma Quality of Life Questionnaire being relatively unresponsive to change.

In COPD, there was a significant increase in pooled quality of life in telehealthcare compared to control, (decrease means improvement, minimally clinical significant improvement −4.0 points) (mean difference −6.6, 95% C.I. −13.7 to 0.5). This meta-analysis pooled only two studies both of which featured an integrated complex intervention with case manager, education, information technology and telephone support. It seems that such intensive interventions were successful.

Reviews often did not report what quality of life scales had been used, but made general statements regarding specific studies: maternal fatigue was reduced by telephone counselling for behaviourally difficult infants and videoconference for parents of very low birthweight infants could improve quality of life by providing emotional support and education to parents [72]. Patients who had Internet access to discussion forums where specialists could respond and feedback to queries had benefits across a range of quality of life outcomes, including higher cognitive functioning, lower negative emotions, more active lifestyle and greater social support [32]. Such benefits became apparent at two months and became sustained after six months.

In diabetes, one review found benefits to quality of life, self-efficacy and social advantages [39], whereas another reported that quality of life was similar in the intervention arm to the usual care arm [41].

The reviews indicated above reported improved quality of life with different forms of telehealthcare. In addition, there was still a substantial minority of reviews in which quality of life was reported as not improved: [36], [43], [50], [86], [93].

### Satisfaction and Other Soft Outcomes

One review compared patients who used telephone contact for consultations with ordinary face-to-face consultations. It found four studies that reported patient satisfaction [22]. Two studies found no significant difference and the other two found that patients who had used telephone contact were more satisfied.

Patients who used telephone follow-up for colorectal cancer were largely satisfied. They also reported reduced travel time and costs and increased convenience [83]. In a large Canadian review of different types of asynchronous telehealthcare it was found that patient satisfaction was generally higher in rural than urban areas and 76% of patients preferred to be assessed by telehealthcare than wait longer for an in-person consultation [69].

Another review of different types of telehealthcare in mixed chronic diseases found that, although clinical outcomes did not improve significantly, patients were overwhelmingly satisfied with the technology. In one study they rated telehealthcare an average of 4 out of 5 on a Likert scale [51].

One review which concentrated on patient satisfaction in telehealthcare for chronic heart failure commented that the description of the definition of patient satisfaction was poor and measured in many different ways with poorly constructed instruments. The authors concluded that this could be improved if interventions were more theoretically-based and standardised and validated instruments in accordance with the American Food and Drug Administration’s recommendations [48].

Patients reported that tele-rehabilitation was convenient and useful [74]. There were also moderate-to-high ratings for participant and parent satisfaction in the review of computerised cognitive behavioural therapy for the prevention and treatment of depression and anxiety in children and adolescents [58].

Overall, it seems that the majority of reviews did not comment on satisfaction, however, where such outcomes had been assessed, it was largely positive. An important criticism raised in one of these revews was that the majority of studies did not define what satisfaction meant and it was not clear if patients were satisfied because the intervention did them no harm or because it was of clear positive benefit. A positive bias may also have been present as a result of the novelty factor of the technology as few studies explored what happened to satisfaction over time [23].

### Safety

There was very little in the systematic reviews specifically concerning patient safety and it was not clear whether adverse events did not occur or whether there was a lack of reporting.

### Costs and Cost-Effectiveness

There was only one review formally examining the cost-effectiveness of telehealthcare [77]. It reviewed the 55 studies found by searching for “cost-effectiveness” in association with “telehealth” in appropriate databases and reported that 24 studies included formal trial data, the remainder being hypothetical or modelling studies. Of these 24 studies, most evaluated cost-effectiveness from the perspective of the health service, while only four undertook an evaluation encompassing the broader societal perspective. Comparisons of the cost of the telehealthcare programmes were absent from two studies and were hypothetical in nine studies. In addition, the authors criticised the techniques used by the majority of studies for calculation of costs and benefits as inappropriate. Overall, cost-effectiveness research was very poor, as it was of small scale and short duration [77]. Many studies included only limited data on financial outcomes and did not fully cost the telehealthcare service [77]. For example, the costs of additional staff required to manage the telehealthcare service were not included. Such additional staff had often clearly contributed to the effectiveness of the telehealthcare intervention [37], [74]. Another review also noted that costs and cost-effectiveness were overwhelmingly considered only from the narrow perspective of the health care provider [72]. In general, the reviews did not provide the evidence to support the optimistic policy rhetoric regarding large potential savings to be enabled by telehealthcare.

## Discussion

### Summary of Findings

The evidence-base represented by existing systematic reviews of telehealthcare research covers diverse types of interventions involving a wide range of outcomes and target groups. The reviews themselves varied in quality. Across the field, the quality of primary studies appears to be generally low, with many studies involving small numbers of patients and lasting for short periods, thus limiting their power to detect meaningful differences in outcomes. Few studies rigorously examined cost-effectiveness whilst even fewer undertook comprehensive economic evaluations that encompassed financial impacts on patients in addition to health services. We did not come across any studies that explicitly examined impacts of telehealthcare on patient safety. Synthesising this evidence base was complicated by the variability in terminologies used to characterise different types of intervention and a general poverty of information about the intervention delivered and its context of use, which muddies the interpretation of results.

Many studies have reported small, statistically significant improvements in surrogate endpoints as a result of telehealthcare interventions, but larger studies of longer duration are needed to determine whether these will be sustainable and translate into improved hard clinical endpoints. There is some evidence that telehealthcare can reduce mortality in chronic heart failure, but no reduction in mortality had been demonstrated for any other chronic condition during the period covered by these reviews. The reduction in heart failure mortality may be due to a general optimisation of medical care in these patients and it is not yet known whether such a reduction persists beyond the first year of telehealthcare. There is some evidence of symptom improvement in depression with tele- cognitive behavioural therapy.

There is stronger evidence that telehealthcare can reduce the frequency of hospitalisation in chronic heart failure, chronic respiratory conditions and diabetes. These improvements appears to be greatest in patients at high risk of mortality and hospitalisation, who may be telemonitored closely to ensure that signs of deterioration are identified early so that steps can be taken to avert the need for admission. Longer term studies are required to see if this prevention of admission will result in reduced mortality or will simply shift the burden of care from hospitals to communities, while the broader economic impacts of prolonging life in high dependency populations is a matter for ethical debate in our aging society.

In some cases, complex telehealthcare interventions included expanded roles for nursing staff in order to reduce the burden on more costly physicians [95]. However, such healthcare professionals require training to support new responsibilities and maintain accountability, and protocols to clarify workflow, each of which may require additional expenditure. This highlights the important costs involved in setting up a new telehealth service, which also include technology purchase, support and service redesign, which may outweigh the economic benefits of reduced hospitalisations or other health service outcomes in the short term. It is arguable that the cost benefit ratio may improve over time as services become embedded and efficiency savings accrue.

Telehealthcare services were usually organised along hospital-style disease definitions, often with a specialised nurse reporting back to a hospital consultant. This has the potential to result in more disease-oriented and less holistic, patient-oriented care, which is problematic in light of the increased prevalence of multi-morbidity with old age [96]. The reviews did not discuss how, for example, a frail elderly patient with more than one chronic illness would navigate between the specialist services available to them.

### Strengths and Limitations

This systematic overview benefits from the breadth of searches undertaken and the depth of the initial conceptual work that informed it [17], [18]. In addition to the usual searches of international medical and scientific databases, databases covering literature from the developing world were searched in order to capture emergent findings from low and medium income settings, although this yielded few results, highlighting the limited coverage and transferability of the existing evidence-base [31], [60]. In contrast to a recent overview by Ekeland et al [97], the data collection in this overview was completed by two reviewers and the data extraction form was piloted several times in several different forms before an optimal form was selected. The quality of the reviews was assessed with reference to a standardised checklist and all review scores were moderated by a second reviewer.

Overviews, or ‘meta-reviews’ such as this are relatively new type of study and attempt to make sense of a broad and deep primary evidence base [98]. The method is most useful for a policy-making audience, in which there is often a need to rapidly grasp key evidential and interpretive information from a broad evidence-base. The technique is inevitably constrained by what is reported in existing reviews and by the time lag between generation of primary literature and secondary literature. In a heterogeneous and fast moving field such as telehealthcare, a balance must also be struck between the desire to capture new publications and the need to appraise them in sufficient detail for meaningful conclusions to be drawn.

Understanding the ‘why’ factors in telehealthcare is critical for informing future programmes, yet many reviews provide only basic descriptions of the telehealthcare interventions, making it difficult to assess which components may have been most critical in determining its success or otherwise. Despite the preliminary work undertaken to define telehealthcare, difficulties arose with inconsistent terminology across reviews and a general lack of precision in the descriptions of telehealthcare in the individual reviews [13]. The implementation of telehealthcare often requires organisational redesign which may exacerbate or reform existing system inefficiencies and uncover new ones. As such, it becomes hard to disentangle the effects of the technology from the human and organisational processes that surround it. In this setting, telehealthcare should be considered as an example of a complex intervention and, as such, may require a more innovative approach to research [99], [100]. A full description of the intervention is an essential part of this, and may need to be combined with qualitative approaches in addition to the classic randomised controlled trial. The technique of “realistic evaluation” – where mid-range theories structured around emergent context-mechanism-outcome (CMO) groupings – are then supported or refuted by further scrutiny of the evidence, holds significant potential for the further understanding of telehealthcare [101], [102], [103], [104].

### Comparisons with Other Literature

Many studies took place with enthusiastic supporters using home-grown technologies and the observed benefits may not therefore be replicable in other contexts, or when scaled-up for use in routine settings [105], [106], [107], [108].

In the UK, the potential benefits of telehealthcare at scale were addressed in the recent NHS Whole System Demonstrator (WSD) Project. This randomised controlled trial incorporated telemonitoring for 6000 patients with COPD, heart failure and other chronic illnesses [109]. The WSD appeared to show significant reductions in mortality and health service utilisation between the intervention and control groups, although later reports indicate that the costs of delivery may have outweighed potential savings [110]. Selection bias may also have influenced the results, as only 20% of those initially interviewed during recruitment to the trial were cleared for participation, while the potential influence of political drivers has also raised questions about the results [111]. A recently published evaluation of quality of life and psychological outcomes reported no benefit of telehealthcare over usual care in patients with heart failure or COPD, although there were also no deleterious effects. [112] An economic analysis revealed an increase in Quality Adjusted Life Years for patients in both trial arms, although the cost of this increase was significantly greater for the telehealth arm, leading the authors to conclude that telehealthcare was not cost effective [113].

Another large trial in the United States, casts some doubt on the positive impacts of telehealthcare in heart failure when implemented at scale. This trial followed over 1600 patients with heart failure randomised between an interactive voice response telemonitoring system and usual care. There was no difference in hospital readmissions or deaths in the groups at six months [106]. A way forward for telehealthcare in heart failure, focusing on personalisation of care and crisis prevention, has been outlined by Anker et al [114].

Largely absent from the systematic review literature on telehealthcare is a consideration of patient preferences. There are policy pressures to shift to lower-cost models of care and this may not suit all patients. Early discussion with patients regarding whether they have the right to refuse telehealthcare and demand a face-to-face consultation may help to avoid possible unintended consequences of service redesign, such as increased emergency department use.

The telehealthcare literature also suffers from the poor representation of particular groups, such as those with multiple comorbidities, cognitive impairment, disabilities or social problems. Although these are often considered in the context of social ‘telecare’, it is important for telehealthcare planners and researchers to also respond to these needs. The equity dilemma is compounded by the so called ‘Digital Health Divide’, whereby socioeconomically disadvantaged groups with the highest health care needs also have poorer technology access and lack the skills to take advantage of eHealth resources [115]. Without addressing this tension there is the risk that implementing telehealthcare may perversely widen health inequalities [116].

We did not find much discussion of data security and privacy issues among these systematic reviews, although we acknowledge that this typically appears in other types of literature. Nonetheless it is important to highlight the potential risks of telehealth data transmission for patient confidentiality and the risks of network vulnerability for patient safety [117], [118], [119].

### Implications for Policy, Practice and Future Research

The findings of this systematic overview raise a number of important considerations for future policy, procurement and research in telehealthcare. The reviews that we have considered encompass a large number of evaluation projects but, like the services they describe, these are rarely scaled to produce large, high quality studies of sufficient duration to determine long term sustainability and impacts. While reported improvements in surrogate clinical endpoints and hospitalisations are encouraging, the evidence overall remains equivocal. Despite the promise that telehealthcare will generate cost-savings for healthcare organisations, governments and society in an ageing population, the economic evidence base is weak and fails to take account of the patient and societal perspective or consider downstream effects on the distribution of care services.

Since the systematic reviews we have included prioritised quantitative evaluations, there was less discussion of sociotechnical factors underpinning successful or unsuccessful telehealth services than appears in other types of telehealth research literature. Introducing telehealthcare typically requires the redesign of services and customisation to suit contextual requirements. There needs to be a thorough consideration of who will be affected and how, and efforts made to predict and mitigate potentially negative unforeseen consequences, along with the provision of adequate resources and plans for evaluation and sustainability. In terms of practice, user-friendly and unobtrusive technology is more likely to be easily implemented [35]. And, critically, there needs to be adequate organisation of workflow to allow quick responses to alerts from the technology alongside planned standard interventions [35].

Future research should include trials with longer-term follow-up and comprehensive economic evaluations in order to evidence the value of telehealth to consumers and health services and to demonstrate return on investment. The randomised controlled trial has the short-coming that it cannot be blinded when delivering telehealthcare and in such circumstances randomised crossover trials may be more appropriate. In order to address the speed of technological change in this area, there is also a need for new evaluative approaches such as “tracker trials” which allow for the evolution of technology projects within the study period in order to maximise the meaningfulness of the results and for informing policies and strategies for implementation [120].

There is also a need for extensive contextual information to be collected and reported on interventions to improve the evidence base. A shared taxonomy for classifying different telehealth interventions, better description of intervention components, and agreement on common outcome measures, assessment tools and metrics, would all help to strengthen the evidence-base by facilitating the synthesis of results across studies and enabling their interpretation with reference to shared and unique factors.

### Conclusions

There is now a very large volume of work investigating the use of a range of telehealthcare delivery models in a number of clinical contexts. Governments and industry have expressed great enthusiasm for these interventions in light of their potential to help manage the increased care demands of an ageing population [9], [10]. On examination, the evidence for favourable impacts on clinical endpoints is modest and measured over the short-term but it appears that telehealthcare can improve outcomes in patients with more severe illness who have most to gain. Telehealthcare also seems to be most successful where the intervention is integrated with education and intensive telemonitoring rather than via simple telephone support. The evidence for cost-savings – the proposition most often used to justify the implementation of telehealthcare – has rarely been generated through robust economic evaluations.

The potential for learning from published studies is also limited due to a lack of emphasis on understanding why interventions succeed or fail. It is critically important to recognise that the term telehealthcare may describe a range of intervention packages implemented in real healthcare organisations and should ideally be studied as part of a complex sociotechnical system. Research should therefore be interdisciplinary and its results used to inform further configuration of services. This is particularly important given the fast pace of technological change in this area, where pressures to adopt new interventions often outpace efforts to accumulate the scientific evidence necessary to demonstrate their value.

## Supporting Information

File S1 **Search Strategy.**
(DOC)

File S2 **CASP Score Tool.**
(DOC)

File S3 **PRISMA Figure.**
(TIF)

File S4 **Studies Figure.**
(TIF)

File S5 **Summary Table.**
(XLS)

File S6 **PRISMA Statement.**
(DOC)
