# Supplementary material for: The Impact of Telehealthcare on the Quality and Safety of Care: A Systematic Overview
Source: PLoS One. 2013 Aug 19;8(8):e71238. doi: 10.1371/journal.pone.0071238 (PMC3747134; doi:10.1371/journal.pone.0071238)
Supplement: File S1 — Search Strategy. (DOC) [file pone.0071238.s001.doc]

**S1 Supplement 1 Search Strategy**

**Search Update: Systematic Reviews, Telehealthcare, Quality and safety**

1. meta-analysis/

2. meta analy$.tw.

3. metaanaly$.tw.

4. meta analysis.pt.

5. (systematic adj (review# or overview#)).tw.

6. exp "Review Literature as Topic"/

7. or/1-6

8. cochrane.ab.

9. embase.ab.

10. (psychlit or psyclit).ab.

11. (psychinfo or psycinfo).ab.

12. (cinahl or cinhal).ab.

13. science citation index.ab.

14. bids.ab.

15. cancerlit.ab.

16. or/8-15

17. reference list$.ab.

18. bibliograph$.ab.

19. hand-search$.ab.

20. relevant journals.ab.

21. manual search$.ab.

22. or/17-21

23. selection criteria.ab.

24. data extraction.ab.

25. 23 or 24

26. review.pt.

27. 25 and 26

28. comment.pt.

29. letter.pt.

30. editorial.pt.

31. animal/

32. human/

33. 31 not (31 and 32)

34. or/28-30,33

35. 7 or 16 or 22 or 27

36. 35 not 34

37. (Wrong site surgery or workaround or underuse or time out or slip$ or side effect$ or sentinel event or safety or safe practice$ or root cause or red rule or read back or quality or proximate cause or production pressure or product recall$ or procedural deviation or overriding alerts or negligence or near miss or misuse or mistake$ or misdiagnosis or medication reconciliation or medical complication$ or leapfrog or adverse drug event$ or adverse event$ or adverse occurrence$ or adverse reaction$ or complication$ or hazard$ or failure$ or incident$ or improve$ or error$ or lapse or information overload or inappropriate or human factors research or human factors engineering or human factors design or human factors engineering or human factors design or human factors or heuristic or harm or face validity or equipment failure or delayed diagnosis or defective product or cost utility analysis or cost benefit analysis or contributing factor$ or confirmation bias or close call or clinical governance or availability bias or appropriate treatment or appropriate care or alert fatigue or adverse drug interaction or iatrogenic or swiss cheese model).tw.

38. ((Diffusion of Innovation/ or Efficiency, Organizational/ or Models, Organizational/ or Organization.mp.) and Administration/) or Organizational Culture.mp. or Organizational Innovation/ or Organizational Objectives/ or Technology Transfer/ or Attitude to Computers/ or Computer literacy/ or Computer User Training/ or Cost Savings/ or Cost-Benefit Analysis/ or (Usability or sustain$ or spread or socio-technical or sociotechnical or implement$ or evaluat$ or computer anxiety or change management or change agent$ or barrier$ or agent of change or adopt$).tw. [mp=protocol supplementary concept, rare disease supplementary concept, title, original title, abstract, name of substance word, subject heading word, unique identifier]

39. Device approval/ or equipment failure/ or equipment failure analysis/ or equipment safety/ or exp health services misuse/ or iatrogenic disease/ or quality assurance, health-care/ or quality control/ or quality indicators, health-care/ or quality of health-care/ or risk reduction behavior/ or software validation/ or equipment design/ or guideline adherence/ or software design/ or program evaluation/ or total quality management/ or (exp medical errors/ not exp observer variation/) or (exp risk management/ not exp risk sharing, financial/) or exp accident prevention/ or (outcome and process assessment).mp. or (Maintenance and Engineering, Hospital).mp. or (Forms and Records Control).mp. or (Facility regulation and control).mp.

40. Electronic Mail/ or ambulatory care information systems/ or home care services/ or telemedicine/ or (telehealth$ or tele-health$ or telemedicine$ or tele-medicine$ or internet$ or computer$ or web$ or telecommunication$ or mobile phone or SMS or tele-monitor$ or telemonitor$ or telemanagement or tele-management or teleconsultation or tele-consultation or telecare$ or tele-care$ or telematic$ or telepharmacy or tele-pharmacy or telenurs$ or tele-nurs$ or video consult$ or email consult$ or e-mail consult$ or remote consult$ or wireless or bluetooth or tele-homecare or telehomecare or remote care or tele-support or telesupport or mobile healthcare or computer mediated therapy or telepathology or tele-pathology or distance technolog$ or remote technolog$ or telepsychiatr$ or tele-psychiatry or teledermatolog$ or tele-dermatolog$ or tele-stroke medicine or stroke thrombolysis or telecardiology or tele-cardiology or telesurgery or tele-surgery or wearable monitor$ or smart home or smart homecare or teleradiolog$ or tele-radiolog$ or distant expert system$ or internet knowledge base or PDA or personal digital assistant or GP to GP or GP2GP or GP2GP or GPtoGP or Web based refer$ or Internet based refer$ or tele-phon$ or telephon$).tw.

41. 37 or 38 or 39

42. 36 and 41

43. 40 and 42

44. limit 43 to yr="2010-Current"
