# Supplementary material for: The Impact of Telehealthcare on the Quality and Safety of Care: A Systematic Overview
Source: PLoS One. 2013 Aug 19;8(8):e71238. doi: 10.1371/journal.pone.0071238 (PMC3747134; doi:10.1371/journal.pone.0071238)
Supplement: File S2 — CASP Score Tool. (DOC) [file pone.0071238.s002.doc]

**S2 Supplement 2 CASP Score Tool**

**Critical Appraisal Checklist for a Systematic Review of Health Informatics Evaluations**

Adapted from:

Critical Appraisal Skills Programme (CASP), Public Health Resource Unit,

Institute of Health Science, Oxford. Oxman AD, Cook DJ, Guyatt GH. Users’ guides to the medical literature. VI. How to use an overview. *JAMA* 1994;272:1367-1371.

REVIEW FOCUS

|  | Yes (+2) | Somewhat (+1) | No or can’t tell (0) |
| --- | --- | --- | --- |
| 1. Did the review address a clearly focussed issue?  Was there enough information on   - The population studied (patients and end-users) - The outcomes considered (how defined, measured etc.) |  |  |  |
| 2. Did the review assess a clearly focussed technology?  Was the technology   - Clearly defined or described - If more than one technology is assessed, were the technologies and there relationship to the other technologies clearly delineated |  |  |  |
| 3. Did the authors look for the appropriate sort of papers?  The ‘best sort of studies’ would   - Address the review’s question - Have an appropriate study design |  |  |  |

VALIDITY OF REVIEW RESULTS

|  | Yes (+2) | Somewhat (+1) | No or can’t tell (0) |
| --- | --- | --- | --- |
| 4. Do you think the important, relevant studies were included?  Look for   - Which bibliographic databases were used - Follow up from reference lists - Personal contact with experts - Search for unpublished as well as published studies - Search for non-English language studies - Comprehensive search string demonstrating awareness of the myriad of MeSH terms available |  |  |  |
| 5. Did the review’s authors do enough to assess the quality of the included studies?  The authors need to consider the rigour of the studies they have identified. Lack of rigour may affect the studies results. Particular attention should be paid to methodological issues surrounding evaluations of health informatics such as unit of analysis and allocation discrepancies, measurement of variables, contamination, transparency of results, etc. |  |  |  |
| 6. Were the studies accurately described?  Such as the functional capacity of the technology(ies), the way in which the end-user interacted with the technology(ies) and degree of compliance, organisational setting and degree of computerisation etc.? When and where the study was conducted and why technology was implemented. Individual study results related back to those elements? |  |  |  |
| 7. Are the results of individual studies reported in a clear and meaningful way or just listed with no real flow?  Consider whether studies with similar characteristics such as organisational setting, outcomes measured and functional capacity of technology(ies) were grouped together |  |  |  |
| 8. If the results of included have been combined, was it reasonable to do so?  (overall result presented from more than one study or meta-analysis)  Consider whether   - The technologies were similar in functionality, integratedness, how output was presented, end-user training, level of compliance, etc - The results were similar from study to study, ie how measured and defined - The results of all the included studies are clearly displayed - The results of the different studies are similar - The reasons for any variations are discussed |  |  |  |
| 9. Did the review demonstrate awareness of its own limitations?  Consider whether the review   - Quality, quantity and consistency of included studies - Presented its findings in light of other similar reviews - Future research indicated? |  |  |  |

RESULTS

|  | Yes (+2) | Somewhat (+1) | No or can’t tell (0) |
| --- | --- | --- | --- |
| 10. Does the review present an overall result?  Consider   - If you are clear about the reviews ‘bottom line’ results, ie is an answer to study question(s) is ascertainable - What these are (numerically or verbally if appropriate) - How were the results expressed (NNT, OR, etc.) |  |  |  |
| 11. How precise are the results?  Are the results presented with confidence intervals if expressed numerically? What words are used to describe effect size? Consistency of findings? |  |  |  |

APPLICABILTY

|  | Yes (+2) | Somewhat (+1) | No or can’t tell (0) |
| --- | --- | --- | --- |
| 12. Implications for policy makers and or those considering implementing such technologies? Appropriate based on findings? |  |  |  |
| 13. Are the results generalisable beyond the confines of the setting in which the work was originally conducted?    Consider whether   - The patients covered by the review could be sufficiently different from your population to cause concern - Your local setting is likely to differ much from that of the review in terms of degree of computerisation and end-user skills, etc - Similar functionality will be employed |  |  |  |
| 14. Were all important outcomes considered?  Such as workflow, patient outcomes, practitioner performance, economic and negative outcomes. |  |  |  |
| 15. Are you able to assess the benefit versus harm and costs?  Even if this is not addressed by the review, what do you think? This is important as A. studies concerning cost-benefit are rarely performed in HI, B. negative outcomes are rarely assessed in studies of HI. Dependent on #14! |  |  |  |
