# Supplementary figures and images for: The Impact of Telehealthcare on the Quality and Safety of Care: A Systematic Overview
Source: PLoS One. 2013 Aug 19;8(8):e71238. doi: 10.1371/journal.pone.0071238 (PMC3747134; doi:10.1371/journal.pone.0071238)

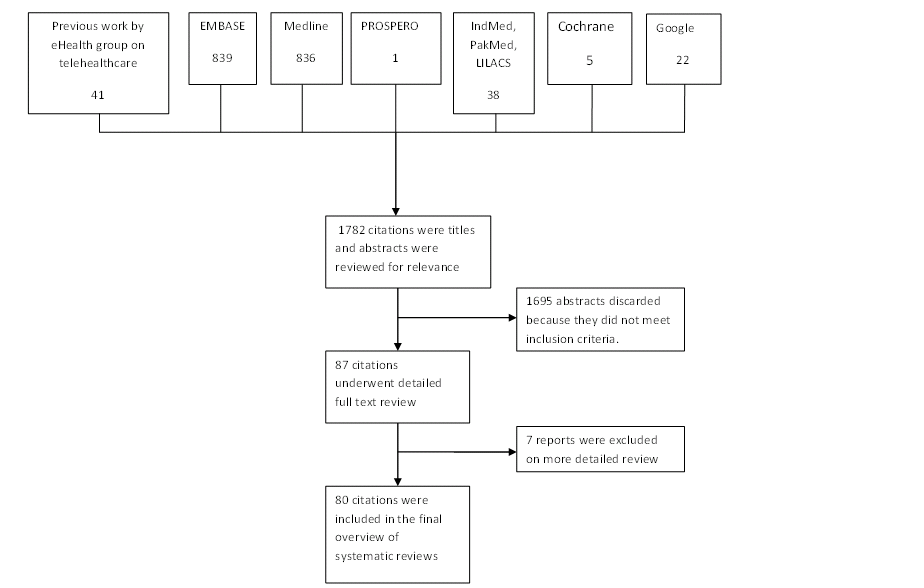

Supplement: File S3 — PRISMA Figure. (TIF) [file pone.0071238.s003.tif]

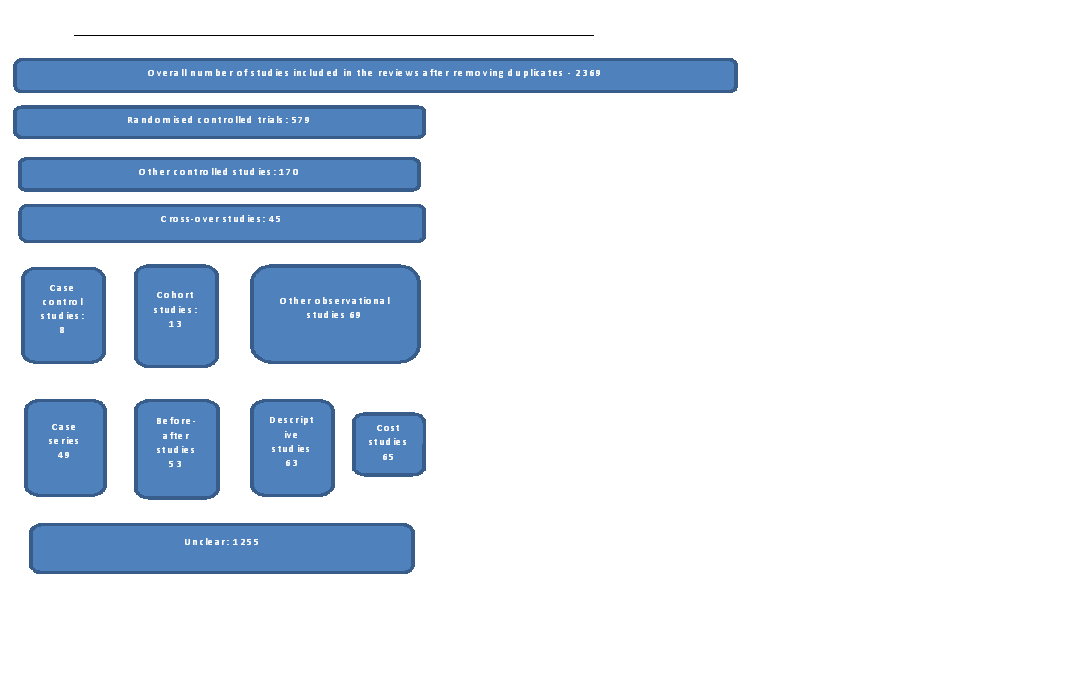

Supplement: File S4 — Studies Figure. (TIF) [file pone.0071238.s004.tif]
